# Feasibility, Acceptability, and Potential Effectiveness of Dignity Therapy for People with Motor Neurone Disease

**DOI:** 10.1371/journal.pone.0096888

**Published:** 2014-05-09

**Authors:** Brenda Bentley, Moira O'Connor, Robert Kane, Lauren J. Breen

**Affiliations:** School of Psychology and Speech Pathology, Curtin University, Perth, Australia; University of Alberta, Canada

## Abstract

**Background:**

Motor neurone disease (MND) practice guidelines suggest developing interventions that will promote hope, meaning, and dignity to alleviate psychological distress, but very little research has been done. This study begins to address this need by exploring the use of dignity therapy with people with MND. Dignity therapy is a brief psychotherapy that promotes hope, meaning and dignity, and enhances the end of life for people with advanced cancer. The aims of this study are to assess the feasibility, acceptability, and potential effectiveness of dignity therapy for people with MND.

**Methods/design:**

This cross-sectional feasibility study used a one-group pre-test post-test design with 29 people diagnosed with MND. Study participants completed the following self-report questionnaires: Herth Hope Index, FACIT-sp, Patient Dignity Inventory, ALS Assessment Questionnaire, ALS Cognitive Behavioural Screen, and a demographic and health history questionnaire. Acceptability was measured with a 25-item feedback questionnaire. Feasibility was assessed by examining the length of time taken to complete dignity therapy and how symptoms common in MND affected the intervention. Generalised linear mixed models and reliable change scores were used to analyse the data.

**Results:**

There were no significant pre-test post-test changes for hopefulness, spirituality or dignity on the group level, but there were changes in hopefulness on the individual level. The results of the feedback questionnaire indicates dignity therapy is highly acceptable to people with MND, who report benefits similar to those in the international randomised controlled trial on dignity therapy, a population who primarily had end-stage cancer. Benefits include better family relationships, improved sense of self and greater acceptance. Dignity therapy with people with MND is feasible if the therapist can overcome time and communication difficulties.

**Conclusions:**

Dignity therapy for people with MND is feasible and acceptable. Further research is warranted to explore its ability to diminish distress.

**Trial Registration:**

www.anzctr.org.au ACTRN12611000410954

## Introduction

Motor neurone disease (MND), also known as amyotrophic lateral sclerosis, is an uncommon neurodegenerative disease that is progressive and always fatal. There is no cure and few options exist for treatment. While a few die within six months, others live ten years or more. On average, people live two to three years after diagnosis before eventually succumbing to paralysis and death, most often from respiratory failure [1], [2].

Despite the considerable physical and emotional suffering involved, there is little focus on addressing the psychological needs of people with MND. Quality of life is generally high [3]–[5], but people with MND often experience significant psychological distress including anxiety and hopelessness [6]. Psychological distress in MND is associated with decreased quality of life and decreased survival rates [7], [8]. Hopelessness is correlated with interest in hastened death [3], [9], [10]. Among those with terminal diagnoses, people with MND report the highest levels of interest in hastened death [11] and they also have the greatest risk of suicide [12]. These effects are mitigated in people who report higher levels of spirituality and sense of meaning [13], [14]. Such findings have led to calls for psychological interventions to bolster hopefulness, spirituality, and meaning in people with MND [1], [15]; however, intervention studies are lacking.

One promising intervention is dignity therapy; a brief psychotherapeutic intervention based on an empirical understanding of dignity at the end of life [16]. Dignity therapy offers people facing death the opportunity to create a document about their life [17]. In a recorded life reflection interview, a person with terminal disease is afforded the opportunity to discuss significant memories, meaningful events and important accomplishments, as well as leave messages for loved ones. In previous studies, dignity therapy has been shown to alleviate existential distress in a palliative care population where most people had malignant conditions [18], [19]. With a key aim to bolster hope and meaning, dignity therapy has the potential to alleviate psychological distress in people with MND [15]. However, because most of the people in previous dignity therapy research had terminal cancer, the findings of its effectiveness are not transferable to people with MND. Diagnosis, ability to communicate, cognitive acuity, stage of illness, baseline levels of distress and demographic features are factors that differentiate people with MND from people with end-stage cancer. Finally, delivery of dignity therapy to people with MND may require modification, for example, to be performed at an earlier time or via assisted communication methods.

### Aims and objectives

The aim of this study was to assess the feasibility, acceptability, and potential effectiveness of dignity therapy to enhance the end of life experience for people with MND. The specific objectives were to determine whether:

- dignity therapy increases hope, meaning, and dignity in people with MND;
- dignity therapy is acceptable to people with MND; and
- it is feasible to provide dignity therapy to people with MND.

## Methods

### Study design

This cross-sectional study utilized a one-group pre-test-post-test design. A control group was not utilized due to 1) the small MND population, 2) access issues to people with MND, 3) ethical concerns over making a potentially useful intervention unavailable to a control group, and 4) the need to test the feasibility of dignity therapy with people with MND [20], [21]. Further details can be found in our protocol [22].

### Ethical approval

This study was approved by the Curtin University Human Research Ethics Committee (19/2011).

### Setting

Participants were primarily enrolled as a result of outreach from the Motor Neurone Disease Association of Western Australia (MNDAWA). MNDAWA sent recruitment letters to people who had been diagnosed with MND and referred to their services by a general practitioner or neurologist. In the last six months of the study, we used social networking, a press release, and information on the university web site to assist in reaching the recruitment goal. One participant in Queensland participated via video-conferencing. Twenty-two participants reported living in an urban/metropolitan area and seven in rural areas. Twenty-seven participants were living at home at the time of the intervention, one in an aged-care facility and one a hospital.

### Participants

Individuals diagnosed with MND, over 18 years old, who could communicate in English and provide informed consent (based on the ALS-Cognitive Behavioural Screen (ALS-CBS) [23] where a cut-off score of 10 was used or the Blessed Orientation Memory Concentration (BOMC) test [24] where a cut-off score of 9 was used) were eligible for the study. Participants were provided with information sheets and written consent was obtained. Enrolment occurred between June 2011 and July 2013. People were excluded if they were too ill to complete the requirements of the protocol. There were no selection criteria based on distress levels, disease stage or proximity to death.

### The intervention

The intervention was administered by a researcher trained in dignity therapy by Harvey Max Chochinov who developed the therapy [18], [19]. The therapy interviews were audio-recorded and transcribed verbatim by a transcriptionist. The researcher shaped the transcribed interviews using the prescribed editing process [17] and then returned to edit and complete the transcripts with the participants. The document was read aloud to each participant at the conclusion of the intervention. To mitigate response bias, post-testing took place via mail or through a visit from a second researcher. The researcher engaged in regular supervision sessions from Prof. Chochinov. To optimize adherence to the dignity therapy protocol, three recordings, transcripts, and completed documents (10%) were reviewed by three experienced researchers (two trained in dignity therapy) and deemed to be adherent.

### Measures and Outcomes

#### Effectiveness

Outcome data to measure potential effectiveness were collected from participants at baseline and one week after completion of dignity therapy. The primary outcome measure was the participant's sense of **hopefulness** assessed with the Herth Hope Index [25], [26], a reliable (α = 0.97) validated instrument developed for use with the terminally ill, with a score ranging from 12–48 and where higher scores indicate more hopefulness. Secondary outcomes were: 1) **Dignity,** measured by the Patient Dignity Inventory (PDI) [27]. The PDI has a scale of 25–125 (higher scores indicate greater distress). It is a reliable (α = 0.93) validated measure which evolved directly from the empirical studies into dignity concerns in the terminally ill. [27] 2) **Spiritual well-being,** measured by the Spiritual Well-Being subscale of the Functional Assessment of Chronic Illness Therapy scale (FACIT-sp-12) [28]. The FACIT-sp-12 has a scale of 0–48 with higher scores indicating greater spiritual wellbeing, and it is a reliable (α = 0.87) and valid measure [28].

#### Acceptability

The Participant Feedback Questionnaire used in the international randomised controlled trial of dignity therapy (IRCT) [19] was modified by adding three items on hopefulness and family support, and was used to collect the participants' experiences and opinions of the intervention. The questionnaire contained 25 questions answered with a 5-point Likert scale and space for brief explanation.

#### Feasibility

Data were collected about the time taken to conduct the therapy sessions, any special accommodations made in the delivery of the intervention, deviations from the dignity therapy protocol, reasons for non-completion, and reasons for attrition.

#### Demographic and health status

Disease specific health-related quality of life was measured with the Amyotrophic Lateral Sclerosis Assessment Questionnaire-5 (ALSAQ-5) where scores range from 0–20 (higher scores indicating more impairment) [29], and cognitive behavioural functioning was assessed with the ALS –CBS [23]. Level of impairment of the person with MND and change in physical function over time was collected from the family carer using the Amyotrophic Lateral Sclerosis Functional Rating Scale-R (ALS-FRS) where scores range from 0–48 (lower scores indicating more impairment) [30], [31]. Demographic data on age, gender, education level, marital status, and health history were also collected.

### Analysis

Data were analysed with generalised linear mixed models (GLMM) as implemented through SPSS's (Version 20) GENLINMIXED procedure. Model parameters were estimated with robust standard errors in order to accommodate potential violations of the model assumptions. Participant was treated as a random effect and Time (pre-test, post-test) was treated as a fixed effect. Age, gender, time since diagnosis, marital status, level of education, and number of days from pre-test to post-test were also treated as fixed effects and analysed individually as potential moderators of the intervention effect. In order to optimise the likelihood of convergence, a separate GLMM analysis was run for each of the three outcome measures. The GLMM maximum likelihood procedure is a full information estimation procedure that uses *all* the data present at *each* assessment point. *All* of the pre-test data and *all* of the post-test data are incorporated into the analysis, which reduces sampling bias associated with participant attrition. GPower (Version 3.1) indicated that 29 participants would be sufficient to capture ‘moderate to large’ (*f* = .28) pre-post changes on the outcome variables. A reliable change (RC) score for each participant [32] was computed to investigate the presence of reliable pre-post change at the individual rather than group level. The RC score is the degree to which the person changes on the outcome variable divided by the standard error of difference between the pre- and post-test scores. When the absolute value of the RC score is greater than 1.96, (Wise [33] has argued that this value can be reduced in some situations), it is likely that the post-test score reflects a *real* or *reliable* change. Descriptive statistics were used to summarize demographic variables and feedback responses.

## Results

### Response rate

MNDAWA distributed recruitment letters to all 147 members diagnosed with MND on three occasions between May 2011 and May 2013. Thirty-five people responded (response rate 24%) and 29 of these people completed the study (completion rate 78%). Those who did not complete include three people who changed their mind before entering the study, two who changed their mind after entering the study, two who died before completion and one who was excluded due to cognitive impairment. While all 29 completed dignity therapy, one did not complete any post-test measures due to illness, one completed the feedback questionnaire but not the outcome measures, and three additional participants did not complete the PDI fully.

### Demographic information

Participants, 20 men and 9 women, ranged from 32 to 81 years of age with almost half between the ages of 60 and 69. Twenty-four were married or partnered. Thirteen reached secondary education; 16 achieved university or postgraduate education. (See Table 1 for more demographic information on the study population).

**Table 1:** Demographic characteristics of study group.

Gender	
Male	20
Female	9
Age	
30–39	1
40–49	1
50–59	4
60–69	15
70–79	6
80–89	2
Marital Status	
Married	24
Widowed	3
Divorced/separated	1
Never married	1
Residence area	
Urban/metropolitan	22
Rural	7
Residence type	
Home	27
Hospital	1
Aged-care facility	1
Presently living with	
Spouse	23
Alone	4
Other	2
Highest level of education attained	
Secondary/high school	13
University/technical	13
Postgraduate	3
Current employment status	
None	22
Full-time	2
Part-time	3
On leave	2
Time since diagnosis	
Less than one year	8
One to two years	9
Two to three years	4
Three to four years	0
More than four years	8
Time since initial symptoms	
Less than one year	2
One to two years	10
Two to three years	5
Three to four years	3
More than four years	9
Prior history of depression (before MND diagnosis)	
Yes	6
No	23
Have you been prescribed medication to help you cope?	
Anti-depressant	7
Anti-anxiety	2
Sleeping medication	1
Anti-anxiety & sleeping medication	1
No	18
Do you consider yourself to be a religious person?	
Yes	8
Somewhat	10
No	11
Do you consider yourself to be a spiritual person?	
Yes	10
Somewhat	14
No	5
Cognitive screening scores	
No impairment	19
Suspected mild to moderate impairment	10

### Baseline levels of impairment and distress

The sample group was moderately impaired (ALS-FRS mean  = 32.61, SD  = 9.76). Scores on the ALSAQ-5 indicate the sample had moderate health-related quality of life (mean  = 9.31, SD  = 3.96). The group was hopeful, had low dignity-related distress, but appeared to be facing some struggles with their spiritual wellbeing (see Table 2 Pre-test scores). The mean total score for spiritual wellbeing was 30.7 (SD  = 10.43) which was lower than in people with cancer (mean  = 38.5, SD = 8.1) [28].

**Table 2:** Mean Pre-test Post-Test Scores on Measures for Hopefulness, Dignity, and Spirituality.

Outcome	Pre-test	*N*	Post-test	*N*
Hopefulness (HHI)	38.76 (5.10)	29	36.61 (6.80)	27
Dignity (PDI)	48.59 (15.45)	29	47.59 (12.91)	24
Spirituality (FACIT-sp-12)	30.72 (10.43)	29	30.92 (9.88)	27

### Effectiveness

Descriptive statistics for the outcome variables are reported in Table 2. There were no significant pre-test post-test changes for hopefulness (*F* [1,54]  = 2.79, *p* = .101, *d* = .46), dignity (*F* [1,54]  = 0.45, *p* = .504, *d* = .20), or spirituality (*F* [1,54]  = 0.01, *p* = .936, *d* = .05). Potential moderators of the intervention effect (age, gender, time since diagnosis, marital status, level of education, and number of days from pre-test to post-test) were individually entered in the regression model in order to determine whether significant pre-test-post-test changes would be observed at certain values of the moderator. There was no significant Moderator x Time interactions for any outcomes (all *p*s >.1).

A reliable change (RC) score for each participant [32]. The results indicate that some individuals showed an improvement in hopefulness, while a quarter showed deterioration (see Table 3). Interestingly, all of the study participants who had an increase in hopefulness reported they were both religious and spiritual, while 43% of the group whose hopefulness declined reported they were neither religious nor spiritual. Additionally, 50% of the group with improved hopefulness had been diagnosed with MND for four years of more, while 85% of the group that declined had been diagnosed for two years or less.

**Table 3:** Percentage (Number) of Participants Showing Reliable Improvement, Deterioration, and No Change for Hopefulness, Dignity, and Spirituality.

Outcome	Improved	Deteriorated	No change	*N*
Hopefulness (HHI)	14.8 (4)	25.9 (7)	59.3 (16)	27
Dignity (PDI)	0	0	100 (24)	24
Spirituality (FACIT-sp-12)	0	0	100 (27)	27

### Acceptability

The participants found dignity therapy to be satisfactory (92.8%), helpful to them (89.2%), helpful to their family (85.2%), and would recommend dignity therapy to others with MND (84%). They reported the strongest positive improvements in the dignity-related areas of looking after unfinished business (67.9%), continuity of self (67.9%) acceptance (64.2%), and role preservation (60.8%). There were lesser improvements in feeling like a burden (28.6%), increased will to live (33.3%), lessened sadness or depression (35.7%), and sense of control (35.7%). Seventy percent reported they felt closer to the people who meant the most to them after dignity therapy, and 63% felt dignity therapy would result in better appreciation in them from their families.

The results of the feedback questionnaire are very similar to the results of the dignity therapy arm in the IRCT which showed that dignity therapy outperformed standard care in a palliative care population where 96% suffered from end-stage cancer [19] (see Table 4). In both studies, people undergoing dignity therapy reported the psychotherapy was helpful to them, improved their quality of life and increased meaning. These findings demonstrate that people with MND experience similar benefits from dignity therapy as reported in previous research with people with cancer [19] (see Table 4).

**Table 4:** Results of the Participant Feedback Questionnaire Compared to Dignity Therapy and Standard Care in the IRCT [19].

	People w/MND (*n = *28*)*	Dignity therapy IRCT (*n* = 108)	Standard palliative care IRCT (*n* = 111)
**DT has been helpful to me**	4.18 (0.72)	4.23 (0.64)	3.50 (1.01)
**DT has been as helpful as any other aspect of my health care**	3.50 (0.88)	3.63 (1.04)	3.27 (1.04)
**DT has improved my quality of life**	3.39 (0.79)	3.54 (0.95)	2.96 (0.96)
**DT has given me a sense of looking after unfinished business**	3.68 (0.61)	3.35 (1.01)	2.86 (1.60)
**DT has improved my spiritual wellbeing**	3.36 (0.68)	3.27 (1.09)	3.00 (1.11)
**DT has lessened my sadness or depression**	3.04 (0.96)	3.11 (1.02)	2.57 (0.92)
**DT has lessened my sense of feeling a burden to others**	2.96 (0.92)	2.81 (0.98)	2.58 (0.95)
**DT has made me feel more worthwhile or valued**	3.50 (0.79)	3.38 (0.93)	3.35 (1.00)
**DT has made me feel like I am still me**	3.71 (0.85)	3.81 (0.85)	3.59 (0.92)
**DT has given me a greater sense of having control over my life**	3.18 (0.77)	3.02 (1.02)	3.16 (1.00)
**DT has helped me to accept the way things are**	3.54 (0.92)	3.39 (1.062)	3.31 (1.01)
**DT has made me feel more respected and understood by others**	3.33 (0.98)	3.16 (0.90)	3.04 (0.98)
**DT has made me feel that I am still able to carry out important tasks or fill an important role**	3.61 (0.99)	3.62 (0.97)	3.48 (1.00)
**I have found DT to be satisfactory**	4.21 (0.69)	4.26 (0.63)	3.80 (0.74)
**DT has made me feel that my life currently is more meaningful**	3.54 (0.69)	3.55 (1.05)	3.19 (1.70)
**DT has given me a heightened sense of purpose**	3.32 (0.82)	3.49 (1.04)	3.20 (0.98)
**DT has given me a heightened sense of dignity**	3.36 (0.87)	3.52 (1.04)	3.09 (1.02)
**DT has made me feel more hopeful**	3.00 (0.86)	N/R	N/R
**DT has lessened my suffering**	3.25 (0.75)	2.86 (1.04)	2.70 (1.02)
**DT has increased my will to live**	2.96 (0.98)	2.94 (1.11)	2.76 (1.04)
**DT has helped me feel closer to people who mean the most to me**	3.63 (0.97)	N/R	N/R
**DT has or will be of help to my family**	4.00 (0.78)	3.93 (0.80)	3.20 (1.00)
**DT could change the way my family sees or appreciates me**	3.48 (1.05)	3.58 (1.01)	2.85 (1.00)
**I would recommend DT to other patients and family dealing with motor neurone disease**	4.04 (0.98)	N/R	N/R

Note: Data are mean (SD). Score 1 is strongly disagree, 2 disagree, 3 neither agree nor disagree, 4 agree, 5 strongly agree. N/R = not reported.

### Feasibility

Dignity therapy for the sample took from three to seven sessions, consistent with the standard protocol [17], [34]. The majority of participants (69%) finished the therapy in four sessions (mean  = 4.14). Four participants (13.8%) completed in the standard of two weeks [19]. The time to completion ranged from 7 to 152 days, with about half completing by 25 days (mean  = 42, *SD* = 36). Reasons for extended completion times included (often in combination) the participants' speech impairment, travel, hospital or respite care admissions, family and employment obligations, and desire for more time to work on the document [35].

Participants' use of various assisted communication methods meant that dignity therapy was successfully completed with six people who, due to MND, had lost the ability to speak. An additional three people had moderate speech impairment and these participants had the three longest completion times (87, 134, and 152 days). One participant with moderate speech impairment completed the intervention using videoconferencing and email.

## Discussion

This is the first study to explore the feasibility of dignity therapy with people with MND and, to our knowledge, the first study of a targeted psychotherapeutic intervention for this population. We expected to detect measureable post-intervention increases in hope, dignity and spirituality at the group level but this did not occur. This may be due to a number of reasons, including the difficulties with demonstrating psychosocial change at the end of life with self-report measures [36], the result of evidence which suggests the benefits of psychosocial interventions at the end of life can most readily be shown in patients who have elevated levels of distress [37], and/or that the outcome measures chosen were not sensitive to the impacts that occurred. Very small pre-post effects were present for dignity and spirituality, and the effect for hopelessness was small to moderate. However, without a control group, we were unable to ascertain whether the intervention had a prevention effect against expected declines in hope, dignity, and spirituality over time as a person with MND deteriorates and approaches death. At the individual level, tentative findings are that dignity therapy may be effective at increasing hopefulness in people who are more spiritual and also in some with advanced disease, as reported in previous research [38].

Nonetheless, the positive results on the feedback survey indicate most people with MND believe dignity therapy to be beneficial. The intervention was found to be overwhelmingly positive. Feedback indicated dignity therapy helped enhance the end of life by supporting the unique identity of the person, helping with acceptance, allaying aftermath concerns, finding meaning and purpose, and improving family relationships which mirrors the previous findings of the pilot study and international randomised controlled trial of dignity therapy performed with people with end-stage cancer [18], [19]. Moreoever, people with MND believe dignity therapy will be of help to their family members after death indicating a potential benefit to family members during bereavement as found in other studies [39], [40]

### Feasibility

Dignity therapy with people with MND is feasible if the therapist can overcome time and communication difficulties, as it takes longer to administer with people who have MND than those with cancer. Therapist time was increased in order to travel to participants in their homes to deliver the intervention (previously completed in palliative care settings [19], [34]) and was compounded for participants in rural areas. Ninety-three percent of participants were in the community rather than an in inpatient or care facility. As such, we were less in control of the schedule. Additionally, for people with speech impairment, dignity therapy was prohibitively difficult and time consuming to perform. Adapted methods appear to present viable solutions to these issues, such as some of the therapy being conducted via email or utilizing videoconferencing.

Unique aspects common to MND, including speech impairment and mild to moderate cognitive impairment, did not detract from the benefits of the therapy. These results indicate that dignity therapy is feasible and acceptable, and it offers potential benefits for people with MND.

### Strengths and limitations

The strengths of this feasibility study were the high response rate, high completion rate, a group representative of people with MND in demographic and health status characteristics, the use of MND-specific cognitive and health status measures, and the measure used to assess acceptability being nearly identical to the one used in the dignity therapy international randomised controlled trial, which allows for comparison. The limitations include inadequate power to discover small effects, mild to moderate levels of distress at baseline, the lack of a control group, and the use of outcome measures not developed or validated for use with people who have MND. The study group may not be representative of the MND population as a whole as those who selected to participate may have been more likely to think dignity therapy would be beneficial.

### Implications for future research

This feasibility study sets the stage for a phase II randomised controlled trial. Potential effectiveness should be further explored through research with people with MND with elevated distress. Research into conducting the intervention via email and through videoconferencing is also indicated. There has been one small study with eight participants showing dignity therapy can be delivered using videoconferencing [41] but a larger study is warranted. Future studies should include hope as an outcome as well as explore the possible relationship between a person's spirituality and changes to hopefulness through dignity therapy.

## Conclusions

Dignity therapy for people with MND is feasible and the unique features of MND, including speech impairment and mild to moderate cognitive impairment can be managed, but the intervention is likely to take a greater length of time to complete compared to previous studies, especially with those individuals experiencing speech impairment who do not utilize assisted communication. Dignity therapy is acceptable to people with MND, who report numerous benefits. Further research is warranted to explore its ability to diminish distress.
